# LINC00511 drives invasive behavior in hepatocellular carcinoma by regulating exosome secretion and invadopodia formation

**DOI:** 10.1186/s13046-021-01990-y

**Published:** 2021-06-04

**Authors:** Xueqiang Peng, Xinyu Li, Shuo Yang, Mingyao Huang, Shibo Wei, Yingbo Ma, Yan Li, Bo Wu, Hongyuan Jin, Bowen Li, Shilei Tang, Qing Fan, Jingang Liu, Liang Yang, Hangyu Li

**Affiliations:** 1 Department of General Surgery, The Fourth Affiliated Hospital, China Medical University, Shenyang, 110032 China; 2 Department of General Surgery, Liberation Army Air Force General Hospital, Beijing, 100142 China; 3 Department of Radiation Oncology, The First Affiliated Hospital, Jinzhou Medical University, Jinzhou, 121001 China

**Keywords:** LncRNAs, Exosome, Multivesicular body, Invadopodia, Hepatocellular carcinoma

## Abstract

**Background:**

Tumor cells are known to release large numbers of exosomes containing active substances that participate in cancer progression. Abnormally expressed long noncoding RNAs (lncRNAs) have been confirmed to regulate multiple processes associated with tumor progression. However, the mechanism by which lncRNAs affect exosome secretion remains unclear.

**Methods:**

The underlying mechanisms of long noncoding RNA LINC00511 (LINC00511) regulation of multivesicular body (MVB) trafficking, exosome secretion, invadopodia formation, and tumor invasion were determined through gene set enrichment analysis (GSEA), immunoblotting, nanoparticle tracking analysis, confocal colocalization analysis, electron microscopy, and invasion experiments.

**Results:**

We revealed that the tumorigenesis process is associated with a significant increase in vesicle secretion in hepatocellular carcinoma (HCC). Additionally, LINC00511 was significantly more highly expressed in HCC tissues and is related to vesicle trafficking and MVB distribution. We also found that in addition to the formation of invadopodia in HCC progression, abnormal LINC00511 induces invadopodia formation in HCC cells by regulating the colocalization of vesicle associated membrane protein 7 (VAMP7) and synaptosome associated protein 23 (SNAP23) to induce the invadopodia formation, which are key secretion sites for MVBs and control exosome secretion. Finally, we revealed that LINC0051-induced invadopodia and exosome secretion were involved in tumor progression.

**Conclusions:**

Our experiments revealed novel findings on the relationship between LINC00511 dysregulation in HCC and invadopodia production and exosome secretion. This is a novel mechanism by which LINC00511 regulates invadopodia biogenesis and exosome secretion to further promote cancer progression.

**Supplementary Information:**

The online version contains supplementary material available at 10.1186/s13046-021-01990-y.

## Background

The tumor microenvironment facilitates tumor occurrence and progression [1]. An increasing number of studies have confirmed that extracellular vesicles carry functional cargoes, such as RNA, proteins, and lipids, which play pivotal roles in cell-to-cell communication [2–6]. It is clear that a large number of extracellular vesicles are released during the occurrence and development of hepatocellular carcinoma (HCC) to participate in tumor progression and metastasis [7–9], but the mechanism of extracellular vesicle secretion much remains unclear. Researchers have studied the generation and secretion of extracellular vesicles, trying to explain the specific mechanism of exosome secretion [6, 10]. Our published review confirmed that the process of exosome secretion mainly involves the multivesicular body (MVB) biogenesis, intraluminal vesicle (ILV) formation, sorting of different cargoes, and the regulation of cargo fate [5]. Specifically, cell membrane endocytosis generates early endosomes, which then internalize and form ILVs via endosomal sorting complex required for transport (ESCRT), which includes HRS, STAM1, TSG101 ALIX, VPS4, and VPS33B to form mature MVBs [11]. It is important that MVBs can target lysosomes for content degradation or target the plasma membrane for exosome release [5]. Current research reveals that MVB-targeted transport involves small GTPases and their complexes in combination with different molecular motor proteins and the microtubule cytoskeleton. Centrifugal and centripetal transport through the traction of the cytoskeleton is a key step in the regulation of MVB fate [5, 12]. Importantly, small GTPases (RAB27A, RAB27B, RAB35, RAB11, RAB7, and RAL-1) play well-established roles in MVB trafficking for exosome release [5, 6, 12, 13]. These key proteins may undergo mutations and abnormal expression during tumorigenesis and development, which may regulate abnormal exosome secretion and play a role in tumorigenesis and metastasis [5, 12–15]. The final key step in achieving exosome secretion involves the formation of SNARE complex intermediates (SNAREpins) to complete MVB docking and plasma membrane fusion [5, 16, 17]. The goal is to extensively study the SNARE proteins involved in exosome docking and fusion, including Syntaxin-4, VAMP3, VAMP7, VAMP8, YKT6, SYX5, SNAP23 and SNAP25 [5, 17]. Among them, VAMP3, YKT6, and SNAP23 play key roles in tumorigenesis and in regulating exosome release [16, 18]. For example, a study showed that pyruvate kinase type M2 promotes exosome release from tumor cells by phosphorylating SNAP23 [18]. Our previous work showed that HOTAIR promotes the colocalization of VAMP3 with SNAP23, which influences SNARE complex formation, leading to MVB fusion with the plasma membrane [16]. Mounting evidences have confirmed that the docking and fusion of MVBs is a complex process in which the RAB protein and SM protein are also involved.

Note that the metastasis of tumor cells is an important sign of tumor deterioration. Tumor cells can not only release exosomes to reorganize the extracellular matrix but also secrete MMP (membrane matrix metalloproteinase) and other metalloenzymes to facilitate migration and invasion [19–21]. Invadopodia, which are specialized F-actin-based structures, play a key role in the process of tumor metastasis [20–22]. Several studies have shown that invadopodia are critical docking and secretion sites for MVBs and significantly induce the secretion of exosomes and MMPs to induce tumor invasion and metastasis [5, 21]. Interestingly, previous studies have revealed that the SNARE complex is involved in invadopodia formation, which indirectly affects the exosome secretion, indicating that there may be a positive feedback mechanism involving the SNARE complex and invadopodia [5, 23]. MiR-612 can regulate tumor metastasis and progression, probably resulting in abnormities in invadopodia structure and function in HCC [24]. The abundance of SNX27 plays an important role in the assembly of recycled MT1-MMP to invadopodia and further promotes breast cancer metastasis [22].

However, few studies have revealed the mechanism by which the abnormal expression of lncRNAs affects exosome secretion in tumor cells. LINC00511 has been widely confirmed to be an oncogene, is abnormally expressed in many tumors and induces the malignant biological behaviour of cancer cells [25–27]. However, the relationship between LINC00511, and exosome secretion and the invadopodia formation in HCC has not yet been studied. Our study first confirmed that the abnormal expression of LINC00511 promotes exosome secretion. Furthermore, we confirmed that LINC00511 induces the generation and distribution of MVBs. Interestingly, we found that LINC00511 dramatically regulates the expression of RAB27B and the colocalization of VAMP7 and SNAP23, which are involved in MVB trafficking and fusion with the plasma membrane, respectively. Importantly, LINC00511 induces the formation of invadopodia by inducing MVB docking and exosome secretion and accelerates the effect of LINC00511 on the invasion and progression of HCC. In conclusion, our study showed that LINC00511 induces the release of exosomes and the formation of aggressive invadopodia in HCC cells, which may provide a new perspective for the study of tumor progression.

## Materials and methods

### Cell culture

The Huh7 and Hep3B cell lines were purchased from Shanghai Gene Chem (Shanghai, China). The Huh7 cell line was routinely cultured in DMEM medium (Biological Industries, Shanghai, China) containing 10% fetal bovine serum (FBS) (P30–3302; PAN Biotech), 100 U/mL penicillin and 100 μg/mL streptomycin. The Hep3B cell lines was cultured in minimum essential medium (MEM) (Gibco, Shanghai, China) containing 10% FBS, 100 U/mL penicillin and 100 μg/mL streptomycin. Cells were maintained at 37 °C under a 5% CO_2_ atmosphere.

### Patients and gene set enrichment analysis (GSEA)

RNA sequencing (RNA-seq) data from liver hepatocellular carcinoma (LIHC) tissue (374 cases) and normal tissue (50 cases) were obtained from The Cancer Genome Atlas (TCGA). The HCC tissue data from TCGA databases were categorized into a LINC00511 high expression group and a LINC00511 low expression group. We performed GSEA using GSEA v3.0 software to analyse vesicle gene signatures and performed functional enrichment analysis.

Tumor tissues and adjacent noncancerous tissues from patients were used for PCR, transmission electron microscopy (TEM), immunohistochemistry and immunofluorescence imaging analysis. All patients had been diagnosed with HCC through pathological examinations. No patients had received chemotherapy or radiotherapy before surgery. The study was approved by the Ethics Committee of China Medical University.

### Plasmid generation, and cell infection

All LINC00511 plasmids and small interfering RNAs (siRNAs) were purchased from GeneChem (Shanghai, China). HCC cells were then transfected with the respective constructs using Lipofectamine™ 3000 (Thermo Fisher Scientific, USA) according to the manufacturer’s instructions. The full-length cDNA sequence of LINC00511 was cloned into the pcDNA3.1 vector (GeneChem Shanghai, China) to construct the LINC00511 overexpression plasmid (pcDNA3.1-LINC00511). The siRNA sequences were as follows: RAB27B siRNA: 5′-CTGGTCCTCCGAGCAAAGAAA-3′ and VAMP7 siRNA: 5′-GGCACAAGUCUCCUUGUAATT-3′.

### Antibodies and reagents

The antibodies used for immunoblotting and immunofluorescence were as follows: Cortactin (H222) (3503; Cell Signaling Technology), VAMP7 (NB100–91356; Novus Biologicals), RAB7 (D95F2) (9367 T; Cell Signaling Technology), RAB27B (13412–1-AP; Proteintech), CD63 (GTX28219; GeneTex), β-actin (20536–1-AP; Proteintech), TSG101(28283–1-AP; Proteintech), CD81(A5270; ABclonal), SNAP23 (ab4114; Abcam), HRP-conjugated anti-rabbit IgG (7074S; Cell Signaling Technology), and HRP-conjugated anti-mouse IgG (7076S; Cell Signaling Technology). The reagent used for immunoblotting was Phalloidin-iFluor 594 reagent (ab176757; Abcam).

### Immunoblotting and co-immunoprecipitation (co-IP)

Immunoblotting analysis was performed as previously described [16, 28]. LINC00511-overexpressing HCC cells were lysed with lysis buffer with protease inhibitors for 20 min on ice. Then, the supernatants were incubated with anti-VAMP7 antibody or IgG followed by precipitation with protein A/G-agarose beads (Santa Cruz Biotechnology, China). The beads were sedimented and washed four times with lysis buffer. The precipitated proteins were analyzed by immunoblotting.

### Real-time PCR

Real-time PCR was performed as previously described [16]. The RT-PCR primers used were as follows: RAB5 forward 5′-AGACCCAACGGGCCAAATAC-3′ and reverse, 5′-GCCCCAATGGTACTCTC-TTGAA-3′; RAB7 forward 5′-CTCATTATCGTCGGAGCCATTG-3′ and reverse 5′-AGTGTGGTC-TGGTATTCCTCATA-3′; RAB11 forward 5′-GCTCGGCCTCGACAAGTTC-3′ and reverse 5′-ACTTATACCACTGCGTCTTCCT-3′; RAB27A forward 5′-GGAGAGGTTTCGTAGCTTAACG-3′ and reverse 5′-CCACACAGCACTATATCTGGGT-3′; RAB27B forward 5′-TAGACTTTCGGGAAA-AACGTGTG-3′ and reverse 5′-AGAAGCTCTGTTGACTGGTGA-3′; and RAB35 forward 5′-TTAAGCTTCGATGGCCCGGGACTACGACC-3′ and reverse 5′-TTGGATCCTTAGCAGCAGCGTT-TCTTTCGTTTACTG-3′. The relative abundance was normalized to that of the endogenous control (GAPDH).

### Exosome preparation

Exosomes were purified through standard differential centrifugation protocols as previously described [28]. In brief, exosomes were purified from the cultured medium at 4 °C by sequential centrifugation steps at 300 g (10 min), 2000 g (20 min), and 10,000 g (30 min) to eliminate cellular debris. The supernatant was filtered through a 0.22 mm filter to remove larger microvesicles (> 220 nm) and then further centrifuged (Optima XPN-100 Ultracentrifuge, Beckman, USA) at 100,000 g (90 min) to to extract exosomes. The exosome pellet was resuspended in PBS, centrifuged at 100,000 g at 4 °C for 90 min, and resuspended in 100 μl of PBS for further analysis. Importantly, the same concentrations of exosomes from the Ctrl-Exo and LINC00511-Exo groups were used in the indicated experiments.

### Inverted 3D collagen invasion assay

Transwell inserts (Millipore, #MCEP24H48) were coated with Matrigel (Corning, New York, USA), and transfected Huh7 and Hep3B cells were seeded on the inverted inserts. Medium containing 2% FBS was added to the lower chambers, and the upper chambers were filled with complete media containing 10% FBS and the chemoattractant EGF (25 ng/ml). The cells invaded the Matrigel plugs for 48 h and were stained with 4 mM calcein AM (Sigma, #17783) for 30 min. Then, the cells were imaged by confocal microscopy, and optical sections were captured at 10 μm intervals with a 10× objective on a Nikon A1R confocal microscope.

### Immunofluorescence and confocal imaging

HCC samples were collected from the Cancer Hospital Affiliated with China Medical University. Fresh tissues were directly and quickly cut into frozen sections. Tissue sections and cells were rinsed three times with PBS and blocked in 2% normal goat serum for 40 min at room temperature, followed by overnight incubation at 4 °C with corresponding primary antibodies or Phalloidin-iFluor 594 reagent (1:1000). The next day, the cells were washed three times in PBS and then stained with the appropriate secondary antibodies (1:100) in PBS for 2 h at 37 °C. Afterward, the sections were washed three times in PBS, and the nuclei were counterstained with DAPI for 5 min at room temperature. Images were captured with a Nikon A1R confocal microscope. The primary antibodies used were anti-Cortactin (H222) (3503; Cell Signaling Technology), anti-VAMP7 (NB100–91356, Novus Biologicals), anti-RAB27B (13412–1-AP; Proteintech) and anti-CD63 (GTX28219; GeneTex).

### Immunohistochemistry staining

Thirty cases of hepatocellular carcinoma and adjacent tumors were collected and fixed and then tissue microarrays were generated. Immunohistochemical staining of RAB27B and SNAP23 was performed according to a previous standard method. Immunohistochemistry (IHC) scores are derived from the intensity of the membrane staining (0–3) × the percentage of positive cells (0, 0%; 1, 1–24%; 2, 25–49%; 3, 50–74%; 4, 75–100%). The final IHC score was determined by the intensity score with the percentage of positive cells, ranging from 0 (the minimum score) to 12 (the maximum score).

### Transmission electron microscopy

Fresh HCC samples were quickly frozen and cut into 1.5 mm × 1 mm × 1 mm samples, stored in glutaraldehyde (protected from light) at 4 °C, dehydrated and embedded. Ultrathin sections of tissues and cells were prepared with a Leica Ultracut UCT equipped with a diamond knife (Diatome). Cells and exosomes were examined as previously described [28]. All samples were examined with an H-7650 electron microscope at 80 kV.

### Fluorescence in situ hybridization (FISH)

The subcellular localization of LINC00511 was measured by FISH technology according to the following instructions. The cells were cultured at 10^5^ cells/well and then fixed using 4% paraformaldehyde. Cells were added to the prehybridization solution and then were hybridized with LINC00511 FISH probes (RiboBio) at 37 °C overnight in the dark. After being washed 3 times with hybrid buffer in the dark, the cells were stained with DAPI for 3 min. The fluorescence was imaged using a Nikon A1R confocal microscope.

### Nanoparticle tracking analysis

Exosomes were resuspended in PBS and analysed with a NanoSight NS300 system (Malvern Instruments, Malvern, UK) as previously described [28].

### Nude mouse xenograft experiments and tumor growth measurement

All animal experiments were approved by the Animal Ethics and Welfare Committee of China Medical University. BALB/c nude mice (4 weeks old ±2 weeks, weighing 20 ± 3 g, female) were purchased from Charles River Laboratories (Beijing, China). The mice were injected with 6 × 10^6^ cancer cells infected with the specific lentivirus or shRNA suspended in 100 μl of PBS and 100 μl of Matrigel matrix (BD Bioscience). The mixed cancer cells were injected into the mice, and tumors were monitored and measured until they reached maximum tumor volumes of 1000 mm^3^.

### Statistical analysis

All data are presented as the mean ± SD of at least three separate experiments and were analysed by SPSS version 17.0 software (USA). The differences between the groups were analysed by Student’s t-tests or analysis of variance. Statistical significance was established at *P < 0.05 and **P < 0.01.

## Results

### LINC00511 was highly expressed and related to vesicle trafficking

Tumor-derived exosomes are involved in tumor progression. Previous studies have shown that the progression of HCC is also closely related to exosomes [9]. Our recently published review suggested that vesicle trafficking was important for cancer progression and tumor metastasis [2, 5].

**Fig. 1:**
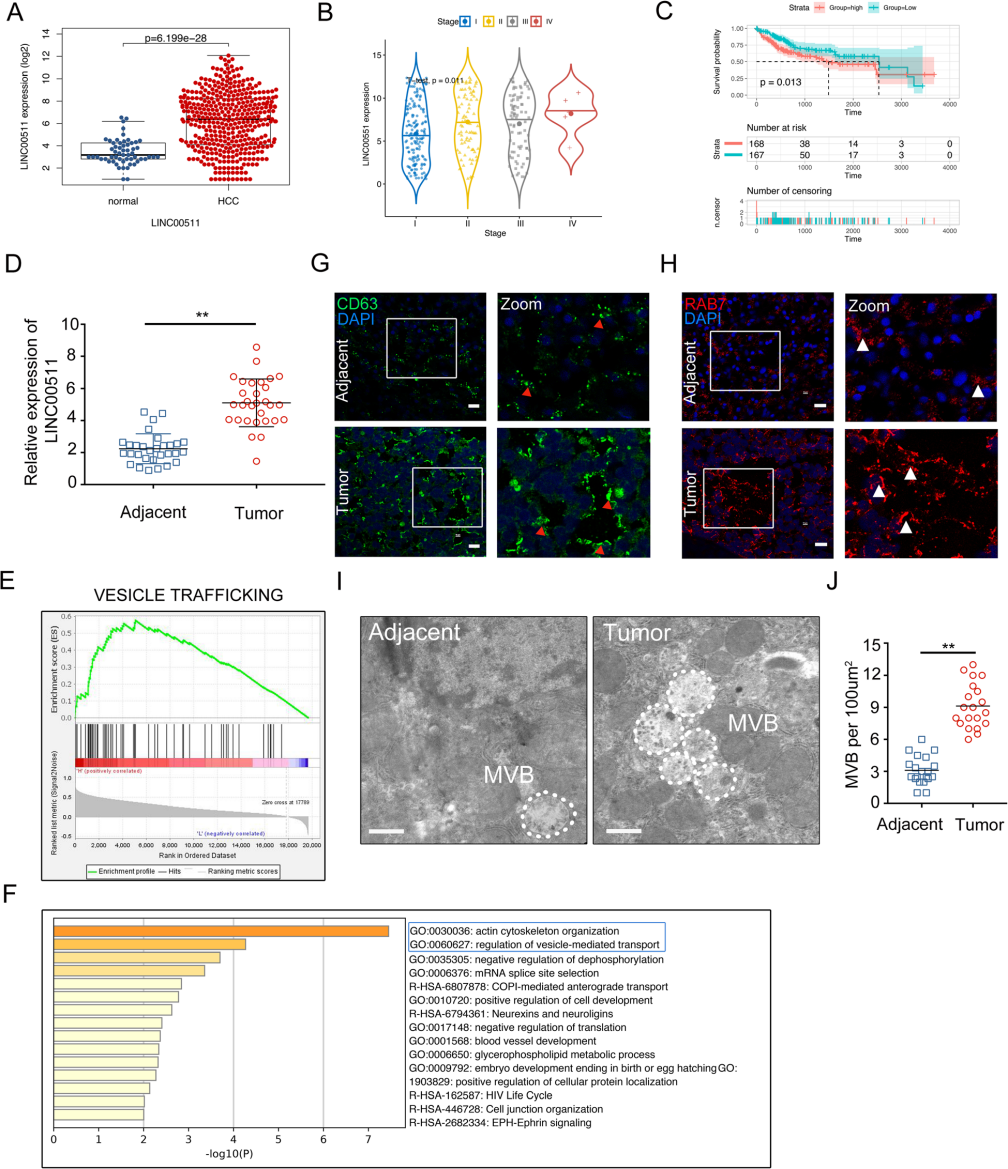
LINC00511 was highly expressed and related to vesicle trafficking. **a** LINC00511 expression in HCC from TCGA (T = 374 cases, *N* = 50 cases). **b** Violin plot shows the relative LINC00511 expression in different stages of HCC in a TCGA HCC cohort (*n* = 374). **c** Kaplan-Meier survival curves generated from TACG cohorts (*n* = 374). **d** LINC00511 expression in HCC patient tumor (T) tissue with paired adjacent non-tumor (NT) samples (*n* = 30). **e** Enrichment plot showing enrichment of vesicle trafficking-related genes in the LINC00511 high expression group. **f** KEGG pathway analysis that the high expression of LINC00511 group was related to vesicle-mediated transport and actin cytoskeleton organization. **g, h** Immunofluorescence of in HCC and adjacent tissues stained with CD63 (green) and RAB7 (red), respectively. Insets show higher magnifications of boxed areas. Arrowheads mark CD63 and RAB7 positive spots. Scale bar, 20um. **i, j** Electron microscopy images showing MVBs in HCC and adjacent tissues. White dotted line indicated MVBs. Scale bar, 500 nm. Quantification of MVBs numbers in HCC and adjacent tissues. Data are mean ± SD from three independent experiments, t-test *, *P* < 0.05, **, *P* < 0.01

To clarify the link between LINC00511 and the secretion of exosomes in HCC, we first examined LINC00511 expression in HCC tissue samples through the TCGA database, and the results showed that LINC00511 was significantly expressed in HCC tissues (Fig. 1a). Furthermore, the HCC stage was significantly positively correlated with the LINC00511 expression (Fig. 1b). Moreover, the prognostic analysis showed that patients with high LINC00511 expression had a significantly poor prognosis (Fig. 1c). We also confirmed that LINC00511 expression was increased in tumor tissue (Fig. 1d), which was consistent with the TCGA database. The results we obtained through the GEPIA database on the LINC00511 expression in HCC, HCC stage and prognosis were consistent with those obtained through the TCGA database (Supplementary Fig. 1a-c). To explore whether the LINC00511 level was related to vesicle trafficking, we first divided the samples into two groups based on the LINC00511 expression level. In the subsequent GSEA, it was clear that the LINC00511 expression was significantly related to vesicle trafficking (Fig. 1e). Further analysis of the KEGG analysis showed that the high expression of LINC00511 group was significantly related to vesicle-regulated transport and the actin skeleton (Fig. 1f).

To clarify the distribution and morphology of MVBs in HCC samples, immunofluorescence analysis was performed and showed that CD63^+^ vesicles and RAB7^+^ vesicles in HCC samples were significantly increased and distributed away from the nucleus (Fig. 1g, h). Moreover, we examined the morphology of MVBs in HCC and adjacent tissues through transmission electron microscopy (TEM). The results showed that the number of MVBs per unit area of HCC tissue was significantly increased (Fig. 1i, j). These results indicate that the tumorigenesis process is associated with a significant increase in vesicle secretion. LINC00511 was significantly more highly expressed in HCC tissues, which is related to vesicle trafficking.

### LINC00511 induces exosome secretion in HCC cells

**Fig. 2:**
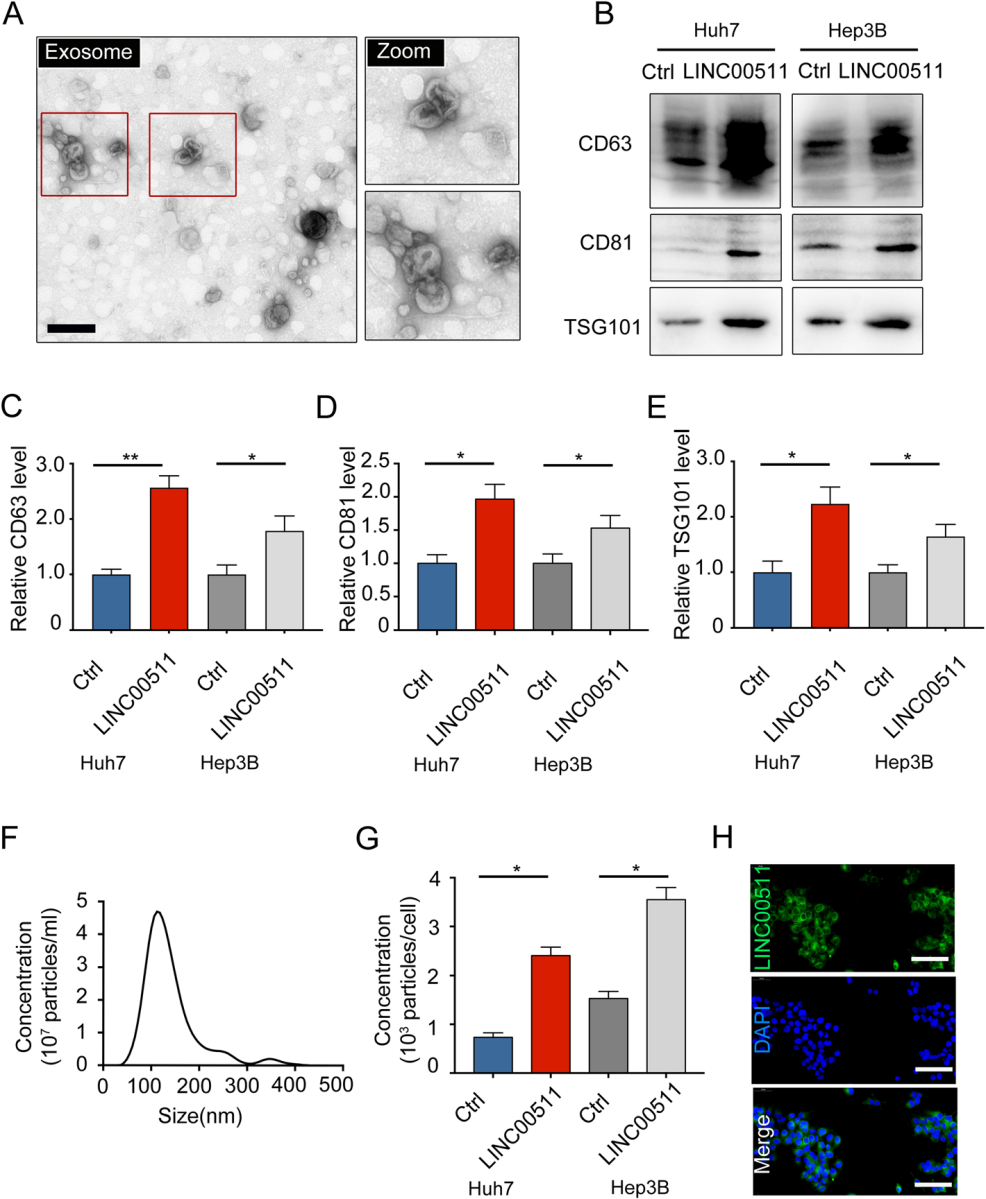
LINC00511 promotes exosome secretion in HCC cells. **a** Exosomes from Huh7 cells assessed by transmission electron microscopy. Scale bar, 200 nm. **b** Exosomes from Huh7 and Hep3B cells transfected with Ctrl or LINC00511 assessed by immunoblotting. Exosomes were analyzed for classical exosome markers CD63, CD81, and TSG101. **c-e** Quantification of CD63, CD81 and TSG101 levels in exosomes isolated from for each condition in (B). The relative intensities were normalized to those from Huh7 and Hep3B cells transfected with Ctrl. **f** The size distribution of exosomes derived from Huh7 cells assessed by nanoparticle tracking analysis (NTA). **g** NTA analysis of the effect of LINC00511 on exosome release in Huh7 and Hep3B cells. **h** LINC00511(green) expresses in the cytoplasm as detected by in situ hybridization FISH in Huh7 cells. Scale bar, 100 μm. Data are mean ± SD from three independent experiments, t-test *, *P* < 0.05, **, *P* < 0.01

To clarify that LINC00511 induces exosome secretion in HCC, we first overexpressed LINC00511 in HCC cells (Supplementary Fig. 2a, b), and exosomes were further purified from the culture supernatants. The TEM results confirmed the structures of classical exosomes (Fig. 2a). Western blotting showed that LINC00511 overexpression significantly induced CD63^+^, CD81^+^, and TSG101^+^ exosome secretion (Fig. 2b). Related quantitative analysis showed that LINC00511 strongly induced the secretion of exosomes (Fig. 2c-e). Subsequently, we further verified that LINC00511 induced exosome secretion through nanoparticle tracking analysis (NTA) (Fig. 2f, g). We also examined the subcellular localization of LINC00511 in HCC through FISH experiments, and the data revealed that LINC00511 was mainly located in the cytoplasm (Fig. 2h). These experimental results confirmed that LINC00511 significantly promoted exosome secretion.

### LINC00511 regulates the biogenesis and distribution of MVBs

**Fig. 3:**
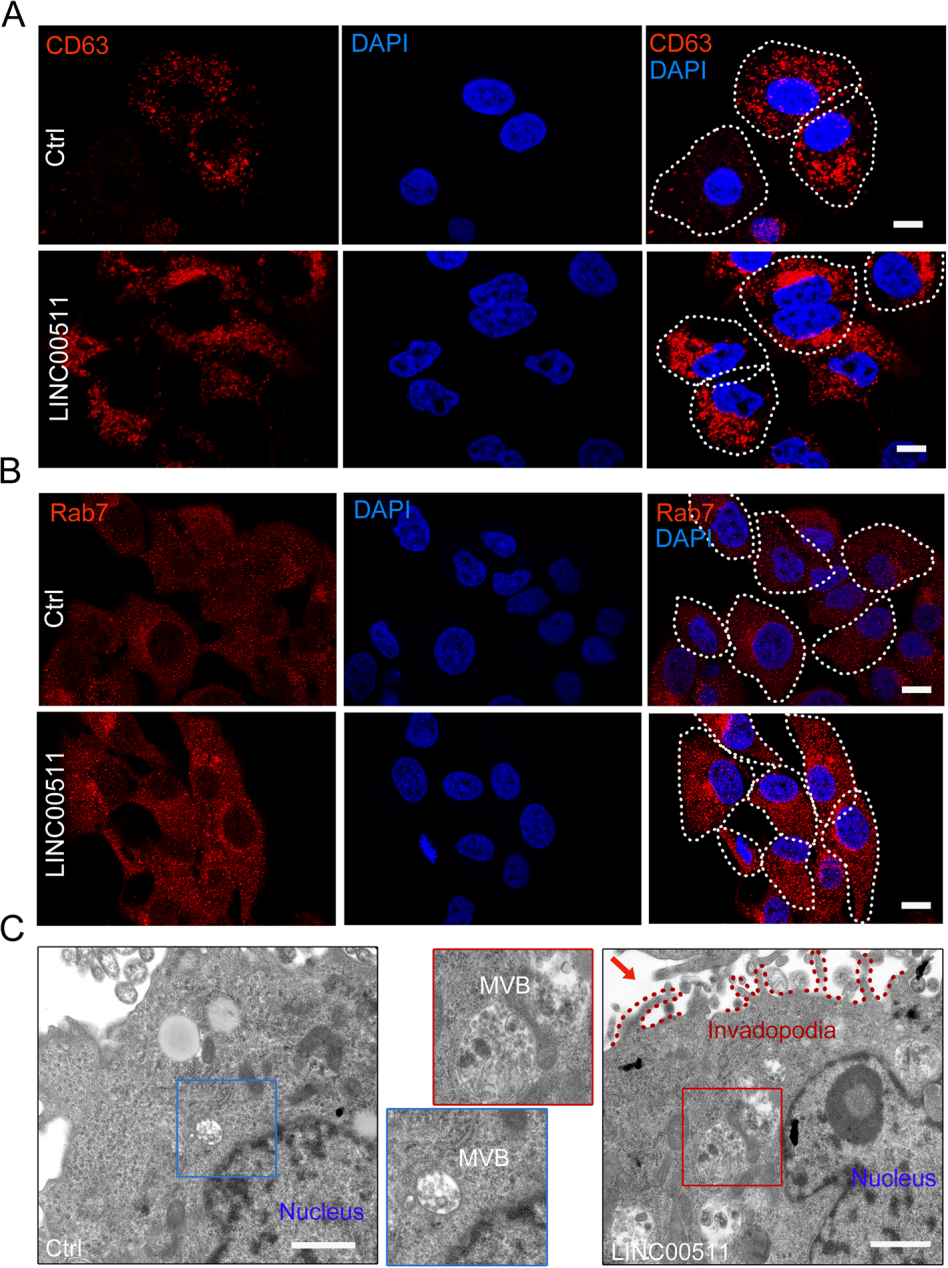
LINC00511 controls the biogenesis and distribution of MVBs. **a, b** Immunofluorescence of cells stained with CD63 (red) and RAB7 (red) in Huh7 cells transfected with Ctrl or LINC00511, respectively. White lines in merged images denote cell periphery. Scale bar, 10um. **c** Electron microscopy images showing MVBs in Huh7 cells transfected with Ctrl or LINC00511.The rectangular box indicates MVBs, and the red dotted line marks the invadopodia structure. Scale bar, 500 nm

Our recently published review revealed that the regulation of MVB biogenesis and targeted transport are the key to controlling exosome secretion [5]. Importantly, we determined that compared with those of adjacent tissues, the number and distribution of MVBs in HCC tissues changed. To explore the mechanism by which LINC00511 regulates exosome secretion, we first investigated MVB changes in LINC00511-overexpressing Huh7 cells. Immunofluorescence analysis suggested that LINC00511 overexpression significantly altered the distribution and number of CD63^+^ vesicles (Fig. 3a, Supplementary Fig. 2c), and RAB7 fluorescence also showed the same results (Fig. 3b, Supplementary Fig. 2). To verify that LINC00511 regulates MVB morphogenesis, TEM experiments were conducted, and the results showed that LINC00511 overexpression markedly increased the number of MVBs in Huh7 cells (Fig. 3c). Interestingly, invadopodia (marked by the red dotted line) were significantly increased in LINC00511-overexpressing HCC cells [21]. In conclusion, our work shows that LINC00511 significantly upregulates the distribution and number of MVBs in HCC cells, further inducing exosome secretion.

### LINC00511 controls MVB targeting to the plasma membrane by regulating RAB27B expression and localization

**Fig. 4:**
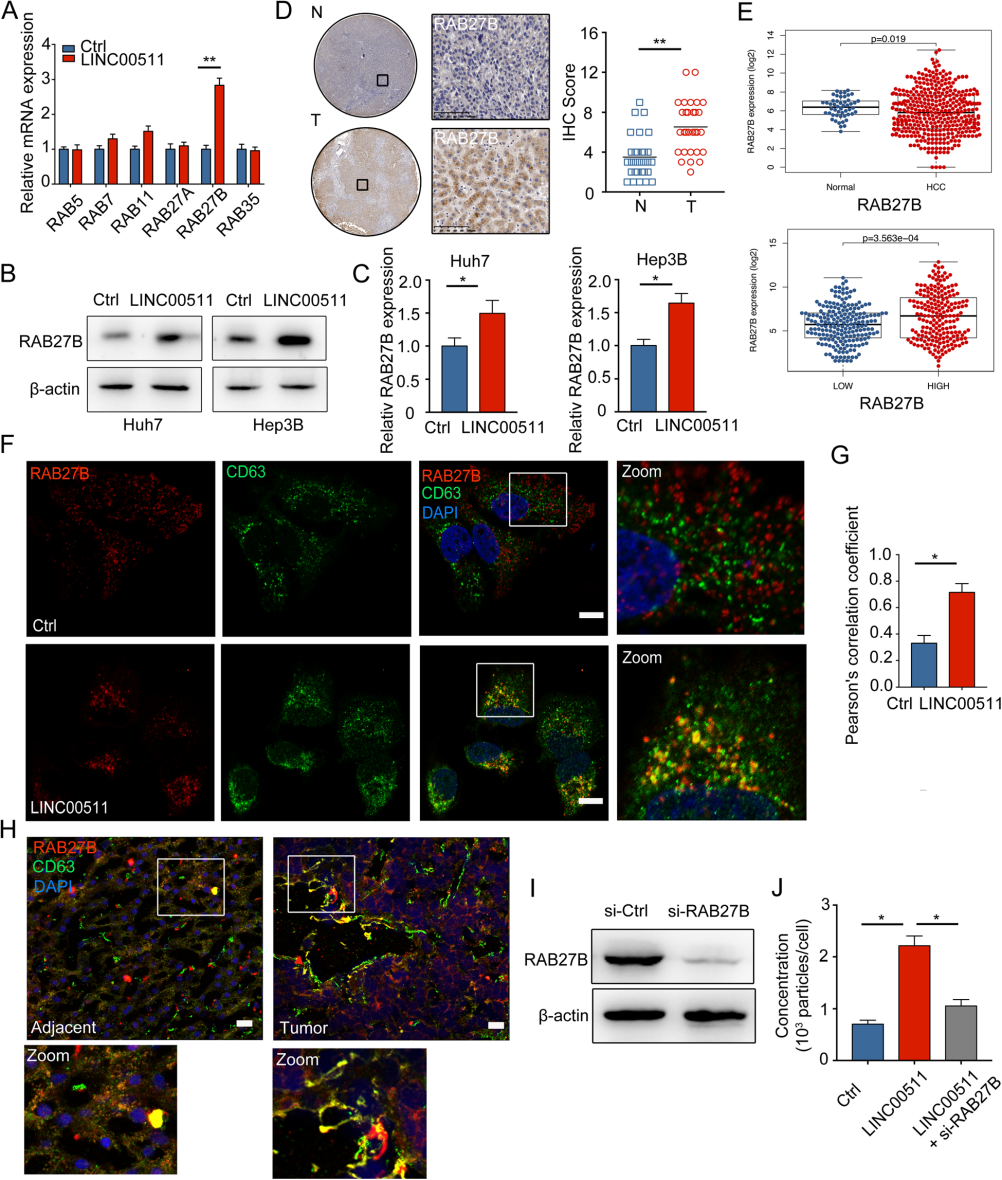
LINC00511 controls MVB targeting to the plasma membrane by regulating RAB27B expression and localization. **a** PCR detected the mRNA levels of RAB5, RAB7, RAB11, RAB27A, RAB27B, and RAB35 in LINC00511 overexpressing Huh7 cells. **b** Immunoblotting analysis of RAB27B expression from the LINC00511 overexpressing Huh7 and Hep3B cells. **c** Quantification of RAB27B levels for each condition in (B). **d** A total of 30 pairs of HCC (T) and para-tumor (N) liver tissues were included. IHC scores for RAB27B were determined. Bars: (left) scale bars, 1.25 mm, (right) scale bars,100 μm. **e** Upper graph: RAB27B expression in HCC from TCGA (T = 374 cases, *N* = 50 cases). Lower graph: RAB27B expression in the high expression of LINC00511 group in HCC from TCGA. **f** Immunofluorescence of CD63 (green) and RAB27B (red) distribution in Huh7 cells transfected with Ctrl or LINC00511. Scale bar, 10um. **g** Colocalization analysis of cells stained for CD63 and RAB27B. **h** Immunofluorescence of CD63 (green) and RAB27B (red) distribution in HCC and adjacent tissues. Scale bars,10um. **i** Immunoblotting analysis of RAB27B expression from transfected huh7 cells with si-RAB27B. **j** Quantification of concentration of exosome release in Huh7 cells co-transfected with LINC00511 and si-RAB27B. Data are mean ± SD from three independent experiments, t-test *, *P* < 0.05, **, *P* < 0.01

It is clear that LINC00511 is involved in the regulation of MVB distribution in HCC. However, the mechanism by which LINC00511 induces MVB trafficking is still unclear. RAB family proteins play well-established roles in MVB transport [29]. RAB proteins bind to different effectors to drive MVB-targeted trafficking [29]. Thus far, RAB27A, RAB27B, RAB35, RAB11, RAB7, and RAB5 have been linked to exosome secretion [5]. Therefore, we performed PCR experiments to determine the mRNA levels of RAB family proteins after LINC00511 overexpression. The PCR results showed that LINC00511 markedly induced the expression of RAB27B (Fig. 4a, Supplementary Fig. 2d). In addition, western blot experiments confirmed that the RAB27B protein level was significantly increased (Fig. 4b, c).

To assess RAB27B expression in HCC tissues, we used immunohistochemistry (IHC) on tissue microarrays (TMAs) from 30 patients with HCC to compare the RAB27B levels in HCC tissues (T) and paired adjacent non-HCC tissues (N). The RAB27B level was significantly increased in HCC tissues (Fig. 4d). Notably, RAB27B expression was also upregulated in HCC tissues in the TCGA database compared with that of normal liver tissues (Fig. 4e). The expression of RAB27B was significantly upregulated in HCC tissues and cells in the LINC00511 group (Fig. 4b, c, e). This finding indicates that LINC00511 can induce RAB27B expression in HCC. The location of RAB27B on the surface of the MVB membrane is key to RAB27B-mediated regulation of MVB transport and further promotes exosome secretion [30]. Confocal fluorescence analysis showed that LINC00511 highly induced the colocalization of RAB27B and CD63 (Fig. 4 f, g, Supplementary Fig. 2e,f). Moreover, compared with those in adjacent tissues, RAB27B and CD63 were also significantly colocalized in HCC tissues (Fig. 4 h). To confirm that RAB27B is involved in LINC00511-regulated exosome secretion, we co-transfected cells with pcDNA-LINC00511 and si-RAB27B (Fig. 4i). The NTA results confirmed that RAB27B regulated exosome secretion in the LINC00511 transfection group (Fig. 4j). Therefore, we suggest that LINC00511 can induce the colocalization of RAB27B and CD63 to regulate plasma membrane targeting to participate in the regulation of exosome secretion.

### LINC00511 is a regulator of invadopodia formation and invasion in HCC cells

**Fig. 5:**
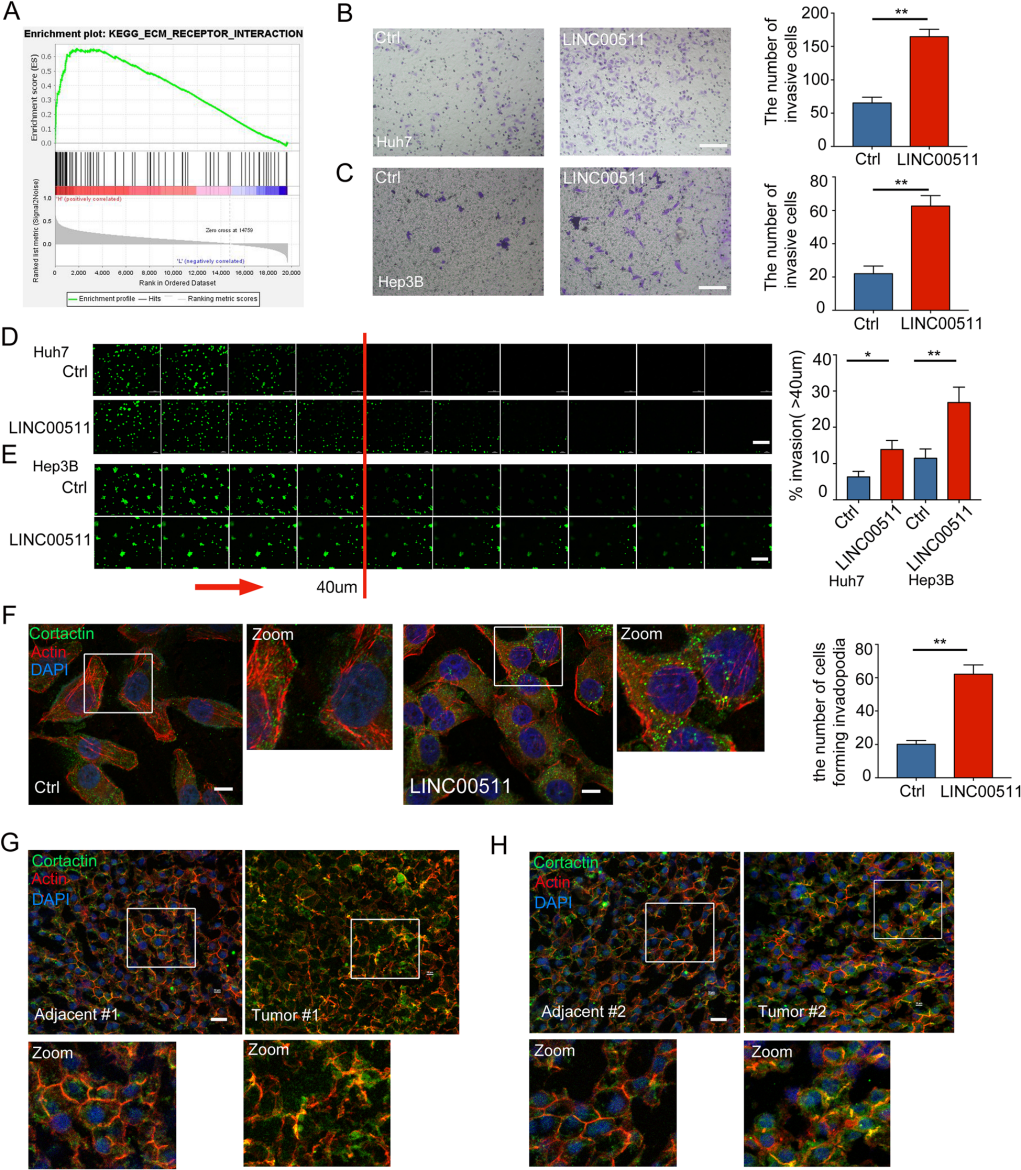
LINC00511 induces hepatocellular carcinoma cell invasion and invadopodia formation. **a** Enrichment plot showing enrichment of ECM-related genes in the LINC00511 high expression group. **b, c** Huh7 and Hep3B cells transfected with Ctrl or LINC00511 were seeded on Matrigel-coated cell inserts and allowed to invade for 24 h, and then invasive cells were counted. Scale bar, 200 um. **d, e** Representative images and quantification of the invasive ability of Huh7 and Hep3B cells transfected with Ctrl or LINC00511. After 48 h of invasion, serial optical sections (10 μm interval) were acquired. Red arrow indicates the direction of invasive movement. Scale bar, 100 μm. **f** Huh7 cells transfected with Ctrl or LINC00511 were stained for invadopodia markers cortactin (green) and actin (Phalloidin-iFluor 594). Images were analyzed on the confocal microscope and the percentage of cells forming invadopodia was quantified and plotted. Scale bar,10 μm. **g, h** Immunofluorescence of in HCC and adjacent tissues stained with invadopodia markers cortactin (green) and actin (Phalloidin-iFluor 594). Scale bar, 20 μm. Data are mean ± SD from three independent experiments, t-test *, *P* < 0.05, **, *P* < 0.01

GSEA showed that LINC00511 is involved in cell invasion (Fig. 5a). Additionally, we conducted cell migration and invasion experiments, and the results showed that LINC00511 significantly induced the invasion of HCC cells (Fig. 5b-e). Studies have demonstrated that the formation of invadopodia by tumor cells is an important step in invasion and metastasis [19, 31]. Interestingly, previous experiments confirmed that invadopodia could drive the secretion of exosomes from tumor cells [20]. In addition, our review described the possible feedback regulation of invadopodia formation and tumor exosome secretion [5, 20]. We used the invadopodia markers F-actin and cortactin to examine the formation of invadopodia in HCC [22, 32]. After LINC00511 overexpression, the colocalization of F-actin and cortactin was analysed by fluorescence confocal microscopy, and the results indicated that LINC00511 significantly induced invadopodia formation in hepatocellular carcinoma cells (Fig. 5f). Further confocal analysis of the morphology and number of invadopodia in HCC tissues showed that the number of invadopodia in HCC tissues was also significantly upregulated (Fig. 5g, h). These results indicate that LINC00511 is involved in invadopodia formation and invasion in HCC cells.

### LINC00511 induces VAMP7-SNAP23 colocalization to mediate invadopodia formation and exosome secretion

**Fig. 6:**
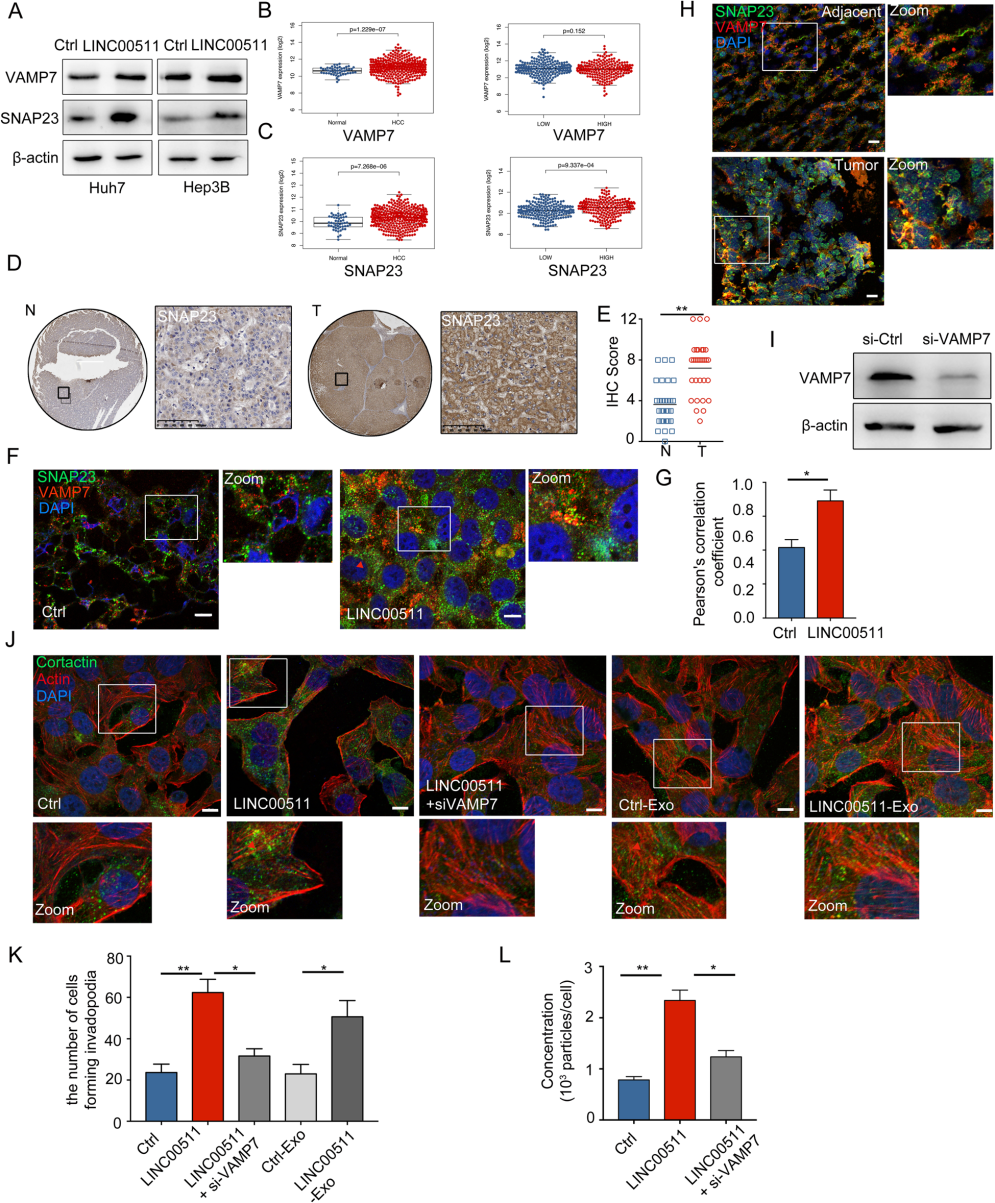
LINC00511 induces VAMP7-SNAP23 colocalization to regulate invadopodia formation and exosome secretion. **a** Immunoblotting analysis of VAMP7 and SNAP23 expression from the LINC00511 overexpressing Huh7 an Hep3B cells. **b** Left graph: VAMP7 expression in HCC from TCGA (T = 374 cases, *N* = 50 cases). Right graph: VAMP7 expression in the high expression of LINC00511 group in HCC from TCGA. **c** Left graph: SNAP23 expression in HCC from TCGA (T = 374 cases, *N* = 50 cases). Right graph: SNAP23 expression in the high expression of LINC00511 group in HCC from TCGA. **d, e** A total of 30 pairs of HCC (T) and para-tumor (N) liver tissues were included. IHC scores for SNAP23 were determined. Bars: (left) scale bars, 1.25 mm, (right) scale bars, 100 μm. **f** Immunofluorescence of VAMP7 (red) and SNAP23 (green) distribution in Huh7 cells transfected with Ctrl or LINC00511. Scale bar,10um. **g** Colocalization analysis of cells stained for VAMP7 and SNAP23. **h** Immunofluorescence of VAMP7 (red) and SNAP23 (green) distribution in HCC and adjacent tissues. Scale bar, 20um. **i** Immunoblotting analysis of RAB27B expression from transfected huh7 cells with si-VAMP7. **j** Huh7 cells co-transfected with LINC00511 and si-VAMP7 or co-culture with Ctrl-Exo and LINC00511-Exo were stained for invadopodia markers cortactin (green) and actin (Phalloidin-iFluor 594). Scale bar, 10 μm. **k** Images were analyzed on the confocal microscope and the percentage of cells forming invadopodia was quantified and plotted. **l** Quantification of concentration of exosome release in Huh7 cells co-transfected with LINC00511 and si-VAMP7. Data are mean ± SD from three independent experiments, t-test *, P < 0.05, **, *P* < 0.01

The last key process of exosome secretion involves MVB docking and plasma membrane fusion [5, 33]. When MVBs are close to the plasma membrane, v-SNARE on the MVB surface and t-SNARE on the plasma membrane pair to form a functional SNARE complex, which induces MVB fusion with the plasma membrane and releases ILVs to form exosomes [5]. The localization of SNAP23 (t-SNARE) on the cell membrane has been confirmed to be involved in the secretion of tumor cell derived exosomes [16, 34]. VAMP7 (v-SNARE) has been confirmed to be involved in membrane transport, cell migration, autophagosome production and other processes. The latest research confirmed that VAMP7 is involved in MT1-MMP^+^ MVB membrane trafficking [23, 35], further regulating the production of invadopodia to maintain the aggressiveness of tumor cells [22, 23, 36]. Western blot analysis confirmed that LINC00511 induced the expression of SNAP23, but not VAMP7 (Fig. 6a). The TCGA database showed the expression of VAMP7 and SNAP23 in HCC and normal tissues (Fig. 6 b, c). Importantly, prognostic analysis revealed that patients with high expression of VAMP7 and SNAP23 had significantly poor prognosis (Supplementary Fig. 3a-b). To examine the SNAP23 expression in HCC tissues, we used IHC analyses of TMAs from 30 patients with HCC to compare the SNAP23 expression in HCC tissues (T) and the paired adjacent non-HCC tissues (N). The SNAP23 level was significantly increased in HCC tissues (Fig. 6d, e). We clarified whether LINC00511 regulates the colocalization of VAMP7 and SNAP23 to participate in the fusion of MVBs and the formation of invadopodia [23]. LINC00511 significantly induced the colocalization of VAMP7 and SNAP23 in HCC cells, as shown by confocal microscopy (Fig. 6f, g). To further clarify the expression and distribution of VAMP7 and SNAP23 in HCC, we used anti-VAMP7- and anti-SNAP23-labelled HCC tissue samples. We found that the expression and distribution of VAMP7 and SNAP23 were different in HCC, and the expression of SNAP23 showed an increase (Fig. 6h). Furthermore, co-IP results showed that VAMP7 binds to SNAP23 in Huh7 cells overexpressing LINC00511(Supplementary Fig. 3c).

These findings indicate that LINC00511 strongly induces VAMP7 targeting to the cell membrane. To further confirm whether VAMP7 is involved in the regulation of invadopodia formation, pcDNA-LINC00511 and si-VAMP7 were co-transfected into Huh7 cells, and the results showed that the formation of invadopodia was significantly reduced (Fig. 6j, k). In addition, our review described the possible feedback regulation of invadopodia formation and tumor exosome secretion [5]. We co-cultured HCC cells with LINC00511-Exo, and the results demonstrated that the formation of invadopodia in Huh7 cells was significantly increased compared with that in the Ctrl-Exo group (Fig. 6j, k). Consistent with this finding, the NTA results suggested that LINC00511 impaired exosome secretion (Fig. 6l). In conclusion, our experiments show that LINC00511 induces the colocalization of VAMP7 and SNAP23 to further induce the formation of invadopodia, thereby strongly inducing exosome secretion.

### LINC00511 regulates the invasion and growth of HCC cells via a reciprocal relationship between invadopodia and exosomes

**Fig. 7:**
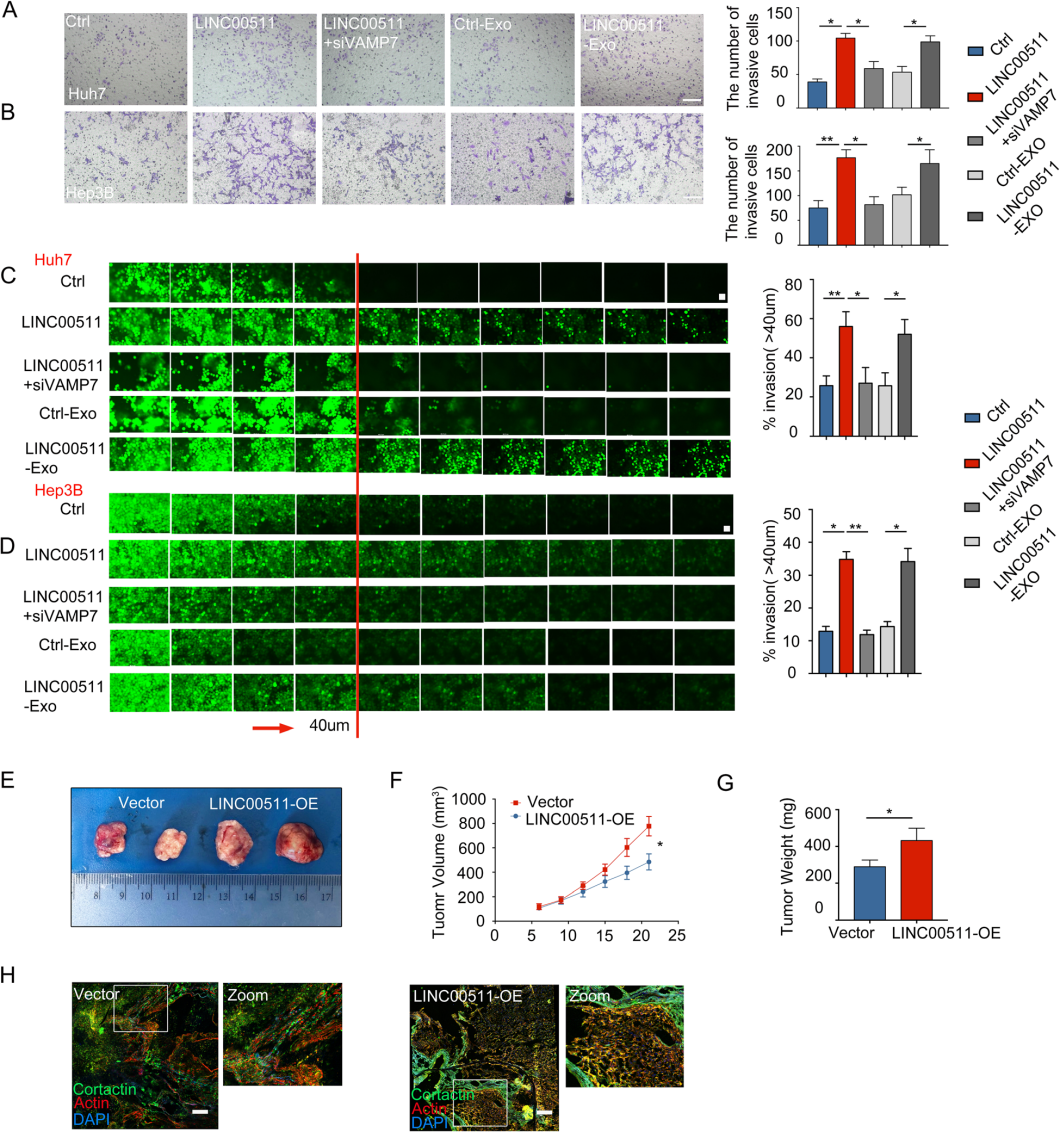
LINC00511 promotes the invasion of HCC cells via a reciprocal relationship between invadopodia and exosomes. **a, b** Huh7 and Hep3B cells co-transfected with LINC00511 and si-VAMP7 or co-culture with Ctrl-Exo and LINC00511-Exo were seeded on Matrigel-coated cell inserts and allowed to invade for 24 h, and then invasive cells were counted. Scale bar, 200 um. **c, d** Representative images and quantification of the invasive ability of Huh7 and Hep3B cells co-transfected with LINC00511 and si-VAMP7 or co-culture with Ctrl-Exo and LINC00511-Exo. After 48 h of invasion, serial optical sections (10 μm interval) were acquired. Red arrow indicates the direction of invasive movement. Scale bar, 100 μm. **e-g** Representative images of the tumors recovered at the end of the experiment. Tumor volume was calculated at the indicated time points and tumor weight was measured in Vector and LINC00511-OE groups at the end of the experiment. **h** Immunofluorescence of tissues from Vector and LINC00511-OE groups stained with invadopodia markers cortactin (green) and actin (Phalloidin-iFluor 594). Scale bars, 100 μm. Data are mean ± SD from three independent experiments, t-test *, *P* < 0.05, **, *P* < 0.01

To understand the importance of LINC00511 in HCC progression, invasion experiments were performed to explore the invasion ability of HCC cells. Our findings confirmed that LINC00511 significantly induced HCC cell invasion (Fig. 7a, b). To clarify the tumor invasion ability, we performed three-dimensional inverted Matrigel invasion assays. The results also showed that LINC00511 markedly induced hepatocarcinoma cell invasion (Fig. 7c, d). Then, si-VAMP7 was used to inhibit invadopodia production and significantly weakened LINC00511-induced cell invasion (Fig. 7a-d). We co-cultured HCC cells with LINC00511-Exo, and the results also revealed that the invasion ability of HCC cells was significantly increased compared with that of the Ctrl-Exo group (Fig. 7a-d). Finally, we performed stable LINC00511 expression experiments in vivo, and the results confirmed that LINC00511 overexpression markedly induced tumor cell growth (Fig. 7e-g). Interestingly, we further confirmed that LINC00511 overexpression significantly induced invadopodia formation, as shown by tissue immunofluorescence (Fig. 7h). Therefore, our results confirmed that LINC00511 regulates the production of invadopodia and the secretion of exosomes, which are involved in the malignant progression of tumors.

## Discussion

**Fig. 8:**
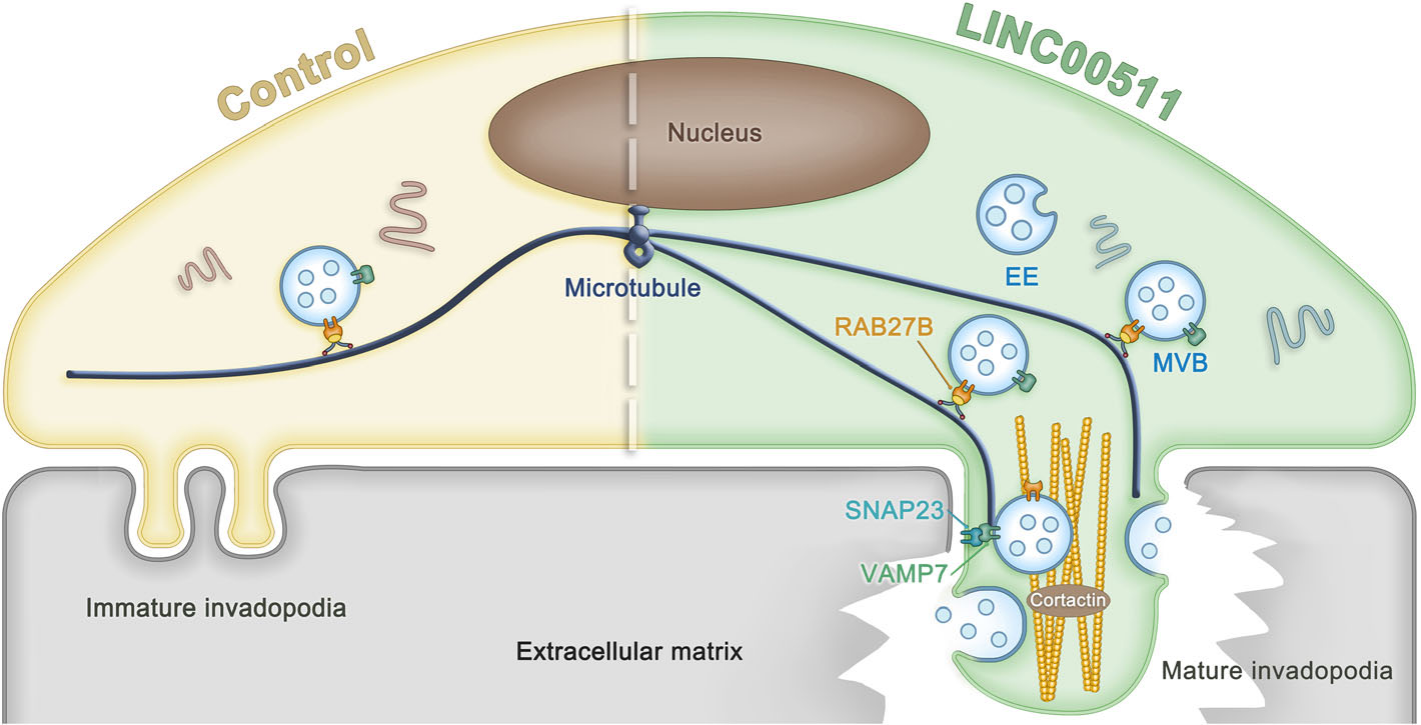
Proposed model for the role of LINC00511 in the regulation of exosome secretion and invadopodia formation, which further facilitating invadopodia membrane extension and extracellular matrix degradation/invasion. LINC00511 induces RAB27B expression and localization to MVBs to drive their anterograde transport, and also induce VAMP7-SNAP23 complex formation to promote exosome secretion, and further promote invadopodia mature and cell invasion. EE, early endosome. MVB, multivesicular body

HCC is a very malignant type of tumor in the digestive tract that has a poor prognosis. Complex high-risk factors and the molecular mechanisms of HCC development and progression remain unclear. In recent years, the involvement of extracellular vesicles in tumor progression and metastasis has been extensively studied. However, the mechanism of EV secretion in tumors is still unknown [4, 7, 37]. Our experiments confirmed that LINC00511 markedly induced the production of MVBs and the release of exosomes in liver cancer and promoted the invasion of HCC. Specifically, we confirmed that abnormally expressed LINC00511 elevates the expression of RAB27B, which induces MVB targeting to the plasma membrane. Furthermore, we also confirmed that LINC00511 induced a marked increase in the colocalization of VAMP7 and SNAP23. We analysed tumor cell invadopodia and further showed that LINC00511 induced invadopodia formation. These findings confirm that LINC00511 regulates MVB docking and plasma membrane fusion to participate in the release of exosomes. Invadopodia formation provides docking and secretion sites for exosomes to further accelerate tumor progression [20] (Fig. 8).

Our previous review discussed the secretion mechanism and fate regulation of MVBs in detail [5]. Importantly, the abnormal expression of genes [38] and mutations [15] in tumor cells, as well as the effects of different stress conditions such as hypoxia [39], pH [40], and the Warburg effect [41], have been reported. These factors are involved in the regulation of MVB fate, thereby maintaining the homeostasis and survival of tumor cells. For example, studies have shown that aerobic glycolysis in tumor cells can promote exosome secretion through the phosphorylation of SNAP23 by its key enzyme PKM2 [41]. Mutant p53 drives cancer cell invasion and metastasis through RAB coupling protein (RCP)-mediated Hsp90α secretion [4]. RCP acts as a critical adaptor protein for the translocation of Hsp90α in the cytoplasm to the endosome for vesicular trafficking and exosome-mediated secretion [4]. However, there are still many unknowns about the regulation of MVB fate.

We found that LINC00511 overexpression induces the distribution and transport of MVBs and exosome secretion. We conducted relevant explorations to confirm that the biogenesis, trafficking and release of MVBs are continuous processes [5]. Many studies have confirmed that many factors participate in the regulation of MVBs and regulate the secretion of exosomes [5]. Among them, RAB proteins are widely localized on the surface of vesicles and participate in vesicle transport by recruiting various effectors [29]. RAB27A, RAB27B, and RAB35 are abnormally expressed in tumors and participate in the regulation of tumor exosome secretion [13, 16, 42]. Among them, RAB27A and RAB27B play different roles in the transport and secretion of MVBs, respectively [30]. RAB27B seems to be more involved in the distribution and movement of MVBs [30]. Our experiments confirmed that LINC00511 overexpression regulates the expression and localization of RAB27B, thereby regulating the targeted trafficking and distribution of MVBs. Meanwhile, research involving the docking and fusion of MVBs and the plasma membrane confirmed that the pairing of v-SNARE on the surface of MVBs and t-SNAREs on the surface of the plasma membrane is the core site of MVB docking and fusion under the action of various auxiliary factors [17]. For example, in endothelial cells, the pairing of vesicle-associated membrane protein 3 (VAMP3) and synaptosomal-associated protein 23 (SNAP23) induces the secretion of microRNA-126-3p-containing exosomes that play a key role in vascular arteriosclerosis [43].

Our previous study confirmed that VAMP3 and SNAP23 on the plasma membrane mediate the release of exosomes in HCC [43]. We overexpressed LINC00511, and it was clear that VAMP3 and SNAP23 did not colocalize in HCC cells (not shown). Interestingly, we found that LINC00511 overexpression markedly increased the colocalization of VAMP7 and SNAP23, which indicates that LINC00511 induces the fusion of MVBs with the plasma membrane by inducing the colocalization of VAMP7 and SNAP23. Karla C. Williams et al. confirmed that VAMP7, SNAP23, and Syntaxin-4 form a SNARE complex that mediates the trafficking of membrane type 1-matrix metalloproteinase (MT1-MMP) during invadopodia formation and tumor cell invasion [36]. Joan Röhl et al. showed that VAMP7 was involved in macrophage MT1-MMP^+^ MVB surface recycling in cancer cells [35], and VAMP7 depletion reduced surface MT1-MMP, gelatinase activity and reduced invasion [22, 35]. Invadopodia are actin-rich plasma membrane protrusions formed by invasive cancer cells that protrude into and degrade the extracellular matrix [20, 22, 44].

In perceiving the microenvironment, tumor cells will form invasive invadopodia that are sufficient to facilitate tumor cell metastasis [31]. Recent studies have shown that invadopodia play key roles at docking and secretion sites for CD63-and RAB27A-positive MVBs [20]. Excitingly, our study showed that LINC00511 overexpression regulated the production of invasive pseudopodia and induced hepatocellular carcinoma cell invasion. When invadopodia formation was inhibited, the colocalization of VAMP7 and SNAP23 induced by LINC00511 and the secretion of exosomes were significantly reduced. Invadopodia biogenesis and matrix-degrading activities are inextricably involved with VAMP7 and SNAP23 complexes [36]. In summary, the SNARE complex is at least partially involved in invadopodia formation and indirectly controls exosome secretion, suggesting that there may be a positive feedback mechanism involving the SNARE complex and invadopodia [5]. Therefore, the formation of invadopodia in cancer cells provides a good site for MVB docking and plasma membrane fusion. Exosome secretion and invadopodia formation accelerate the invasion and migration of cancer cells.

## Conclusion

In summary, we showed that LINC00511 regulates the fate of MVBs and exosome secretion and induces the progression of HCC in vivo and in vitro. Specifically, we suggest that LINC00511 induces MVB trafficking to the plasma membrane by regulating the positioning of RAB27B and MVBs and VAMP7-SNAP23 complex formation. These processes may further induce invadopodia production to accelerate MVB docking and exosome secretion. We also confirmed that invadopodia formation induced by LINC00511 supports ECM degradation and tumor invasion. In conclusion, we propose a mechanism by which LINC00511 induces the release of exosomes and promotes tumor progression.

## Supplementary Information

**Additional file 1: Fig. S1. a** LINC00511 expression in HCC from GEPIA (T = 369 cases, *N* = 160 cases). **b** Violin plot shows the relative LINC00511 expression in different stages of HCC from GEPIA (*n* = 369). **c** Kaplan-Meier survival curves generated from GEPIA (*n* = 364). *, *P* < 0.05.

**Additional file 2: Fig. S2. a, b** PCR detected the LINC00511 expression in Huh7 and Hep3B cells transfected with Ctrl and LINC00511. **c** Immunofluorescence of cells stained with CD63 (green) and RAB7 (green) in Hep3B cells transfected with Ctrl or LINC00511, respectively. Scale bar, 10um. **d** PCR detected the mRNA levels of RAB5, RAB7, RAB11, RAB27A, RAB27B, and RAB35 in LINC00511 overexpressing Hep3B cells. e Immunofluorescence of CD63 (green) and RAB27B (red) distribution in Hep3B cells transfected with Ctrl or LINC00511. Scale bar, 10um. f Colocalization analysis of cells stained for CD63 and RAB27B. Data are mean ± SD from three independent experiments, t-test **, *P* < 0.01.

**Additional file 3: Fig. S3. a** The association of SNAP23 expression with HCC prognosis, kaplan-Meier survival curves generated from TACG cohorts (*n* = 374). **b** The association of VAMP7 expression with HCC prognosis, kaplan-Meier survival curves generated from TACG cohorts (*n* = 374). **c** Huh7 cells were transfected with LINC00511. The cells were lysed 48 h after transfection and immunoprecipitated using anti-VAMP7 antibody or IgG. The association between VAMP7 and SNAP23 was determined by immunoblotting with the SNAP23.

## Data Availability

Other datasets analyzed during the study are available from the corresponding author on reasonable request.

## Abbreviations

MVB: Multivesicular body
ESCRT: Endosomal sorting complex required for transport
VAMP7: Vesicle associated membrane protein 7
RAB: Ras-related protein
SYX5: Syntaxin 5
HCC: Hepatocellular carcinoma
LncRNAs: Long noncoding RNAs
GSEA: Gene set enrichment analysis
TSG101: Tumor susceptibility gene 101
ALIX: Programed cell death 6-interacting protein
CD63: Cluster of differentiation 63
SNARE: Soluble N-ethylmaleimide-sensitive fusion factor attachment protein receptor
PBS: Phosphate-buffered saline
MMP: Membrane matrix metalloproteinase
SM: Sec1/Munc18
